# Native Prey and Invasive Predator Patterns of Foraging Activity: The Case of the Yellow-Legged Hornet Predation at European Honeybee Hives

**DOI:** 10.1371/journal.pone.0066492

**Published:** 2013-06-18

**Authors:** Karine Monceau, Mariangela Arca, Lisa Leprêtre, Florence Mougel, Olivier Bonnard, Jean-François Silvain, Nevile Maher, Gérard Arnold, Denis Thiéry

**Affiliations:** 1 UMR 1065 Santé et Agroécologie du Vignoble, INRA, F-33883 Villenave d’Ornon, France; 2 Université de Bordeaux, ISVV, UMR 1065 Santé et Agroécologie du Vignoble, Bordeaux Sciences Agro, Villenave d’Ornon, France; 3 CNRS, Laboratoire Evolution, Génomes et Spéciation, UPR 9034, CNRS, 91198– Gif-sur-Yvette, France and Université Paris-Sud 11, Orsay, France; 4 Unité de Recherche IRD 072, Laboratoire Evolution, Génomes et Spéciation, UPR 9034, CNRS, 91198– Gif-sur-Yvette, France and Université Paris-Sud 11, Orsay, France; University of Arizona, United States of America

## Abstract

Contrary to native predators, which have co-evolved with their prey, alien predators often benefit from native prey naïveté. *Vespa velutina*, a honeybee predator originating from Eastern China, was introduced into France just before 2004. The present study, based on video recordings of two beehives at an early stage of the invasion process, intends to analyse the alien hornet hunting behaviour on the native prey, *Apis mellifera*, and to understand the interaction between the activity of the predator and the prey during the day and the season. Chasing hornets spent most of their time hovering facing the hive, to catch flying honeybees returning to the hive. The predation pressure increased during the season confirming previous study based on predator trapping. The number of honeybee captures showed a maximum peak for an intermediate number of *V. velutina*, unrelated to honeybee activity, suggesting the occurrence of competition between hornets. The number of honeybees caught increased during midday hours while the number of hornets did not vary, suggesting an increase in their efficacy. These results suggest that the impact of *V. velutina* on honeybees is limited by its own biology and behaviour and did not match the pattern of activity of its prey. Also, it could have been advantageous during the invasion, limiting resource depletion and thus favouring colonisation. This lack of synchronization may also be beneficial for honeybee colonies by giving them an opportunity to increase their activity when the hornets are less effective.

## Introduction

A prey-predator system is maintained when both prey and predators develop strategies that allow its durability. Natural selection should thus favour prey able to avoid their predators. Several processes lead to efficient predator avoidance such as the detection based on odour [1], [2] or on visual cues [3], the secretion of chemical compounds to repel or deter predators [4], [5], morphological traits (e.g. neckteeth in *Daphnia sp.* [6]), warning signals (including behaviours, sounds and colours, i.e., aposematism) or alternatively crypsis [7]–[11], and/or direct behavioural response (fleeing [12], feigning death [13], [14], attacking [15]–[18]). Conversely, predators are selected for their efficacy to acquire prey, i.e., in bypassing their defence [19]–[24].

Another efficient antipredator behaviour consists in reducing the risk of capture by temporal and/or spatial predator avoidance. Predator hunting activity depends either on their own biology or fits their prey activity [25], [26] and conversely, the prey might adjust its activity according to the occurrence of its predator in the environment [25]–[30]. Consequently, different patterns of activity can be observed. *First*, the predator activity can match the activity of its prey, i.e., activity patterns are synchronous. In this case, the predator enhances its performance and reversely the prey has to deal with high predation risk. *Second*, the prey avoids the predator maximum activity period to reduce the predation risk, i.e., activity patterns are decoupled and opposed. *Third*, the predator activity is driven by its own biological rhythm independently of the prey activity pattern, i.e., activity patterns differ but partially overlap. In this case, the predation risk for the prey and the success of the predator are intermediate.

Invasive alien species are considered to be one of the major causes of biodiversity loss worldwide [31]–[33]. Especially, the introduction of alien generalist predators may impact the food-web on the whole ecosystem in changing inter-specific relations through predation and/or competition [34]–[39]. Contrary to prey-predator co-evolutionary systems, alien predators may benefit from the naïveté of their prey and thus have a higher impact on them than the native predator species would have [35], [40]–[44] (but see [30] and [45]). However, the impact of the invasive predator on its native prey is also mostly determined by the temporal overlap between their respective patterns of activity. Thus, monitoring prey and predator activity patterns could help in understanding the mechanism of a successful biological invasion and assessing the risk for prey populations [30], [46]. At an early stage of the co-evolutive process, the prey may not be able to avoid its predator; thus decoupled and opposed patterns of activity are less likely to occur (but see [30]). Alternatively, one may expect that the invasive predator and the native prey may have either synchronous patterns of activity if the predator is able to fit its prey activity or patterns of activity that partially overlap [46].

Social wasp species are generalist foragers hunting a wide spectrum of arthropod prey including other social hymenopterans [47]–[49]. During the last century, they were responsible for several biological invasions worldwide [50], [51], principally favoured by their social organisation, their cognitive and communication abilities allowing behavioural plasticity [35], [52]–[54]. Europe faces its first hornet invasion, which started several years ago [51], [55]. *Vespa velutina*, the yellow-legged hornet, is a generalist predator first observed in France in 2004 and originating from eastern China [56]. Workers prey on several arthropod species in order to feed larvae but so far, most of this predation has been directed towards domestic honeybees. In its native area, *V. velutina* hunts the native and the introduced honeybee species, *Apis cerana* and *A. mellifera* respectively. *Apis cerana* which is supposed to have co-evolved with *V. velutina* is able to exhibit efficient antipredator behaviours against this hornet species whereas *A. mellifera* is less defensive thus suffering a higher predation pressure [10], [11], [15]–[18]. The introduction of this hornet species into France generates the reversed situation, *A. mellifera* being the native prey and *V. velutina* the alien predator.

Interestingly, studies involving *V. velutina* have been mainly focused on the defensive behaviours of *A. mellifera* and *A. cerana* such as the bee-carpet occurrence, the “balling” behaviour and/or the abdomen shaking movement [10], [11], [15]–[18], [56]. Neither the behaviour of the invasive population of *V. velutina* nor the temporal patterns of the prey-predator interaction has been investigated to date. In the present study, we first described the hunting behaviour of *V. velutina* based video monitoring of colonies during an entire predation season in 2009, i.e., the fourth season after the first detection of *V. velutina* in this area. Then, we analysed the diurnal and seasonal variations of the hornet and the honeybee activity to know how prey and predator patterns of activity vary throughout the season.

## Materials and Methods

### Ethics Statements

No permits were required for the described study, which complied with all relevant regulations.

### Study Location

This study was performed in an experimental apiary (INRA, Villenave d’Ornon, France, GPS: N 44°47′31.26" W 0°34′29.99") located in an area where *V. velutina* predation on honeybees has been first reported in 2005 and lasts from early July to November [57]. The experiment was conducted four years after the area was invaded. In 2009, at least six *V. velutina* nests were detected within 1 km of the hives but their respective contribution to the local predation is unknown. Two *Apis mellifera* hives (thereafter called H1 and H2) were video monitored from June to November (see below). The hives were of similar appearance. The exact size of the honeybee colonies was unknown but potential differences were controlled in the statistical analyses (see *Statistical analyses* section for details).

### Video Recording

**Figure 1:**
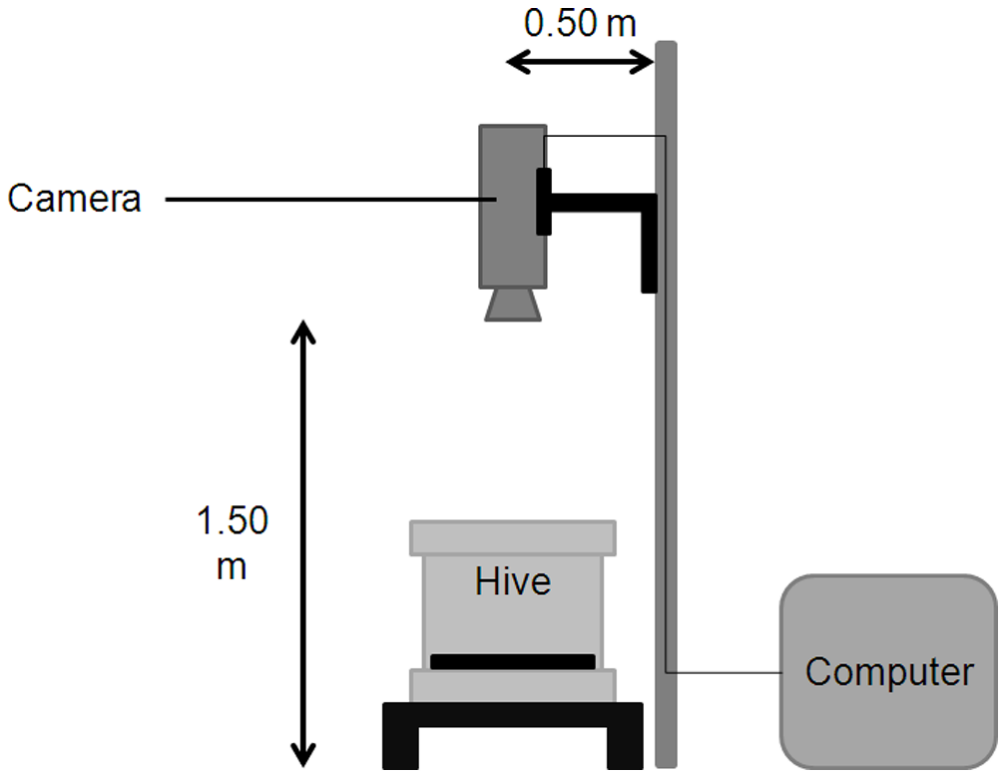
Position of the video camera above the entrance of each hive.

*Vespa velutina* and *Apis mellifera* being diurnal, video recording was programmed with a digital recording software (Numeriscope, Viewpoint, France) to begin at sunrise and to stop at sunset on the two hives. Recording began on the 19/06/2009 (before the first observation of a hunting hornet) and ended on the 23/11/2009. Video cameras (black and white Dragonfly Point Grey, 640×480 resolution, 100 FPS) connected to a computer (for video storage) was fixed on a mast, 1.50 m above the ground, at a distance of about 0.50 m from the hive (Fig. 1). In such a position, the camera does not disturb honeybees and hornets. To have a good detection of the insects, the soil below the landing board was covered with a homogeneous plywood board.

### Video Analysis

From the 19/06/2009, videos were visualized in order to detect the occurrence of the first *V. velutina*: the video analysis started at this first occurrence on the 10/07/2009. We determined the last day of the analysis (25/10/2009) by the absence of honeybees on the hive entrance and the presence of hornets inside the two hives (as well as the following days). Over this period, one sample day was analyzed every 10 days, except for the second date (20/07/2009) postponed five days (25/07/2009) and the third date (04/08/2009) postponed two days (06/08/2009) because of bad weather conditions. Overall, 11 sample days were used for the analyses. For each sample days, sample points were realized at the beginning of each hour, every hour during daylight accordingly with season variations. Two sample points at 08∶00 am (05/10/2009 and 15/10/2009) were not included in the analyses because of little visibility on videos. Our total sample for the whole observation period consisted in 139 sample points per hive (mean = 12.64 sample points per day per hive, range: 10–14).

We chose to monitor *V. velutina* behaviour during the first five minutes of each hour. The number of *V. velutina* was assessed by the maximum number of hornets present at the same time during the five minute sample point and the number of honeybees captured and their location (flying in or out the hive, on the hive walls or on the ground).

We defined two positions around the yaw axis for hornets in their characteristic stationary flight: *(i)* facing the hive (thereafter called “front position”) when the hornets were directed in a 180° sector toward the hive entrance and *(ii)* away from the hive entrance (thereafter called “back position”) when the hornets were directed in a 180° sector away from the hive entrance. We quantified the time spent by the hornets either in front or in back position once per sampled day at 2∶00 pm which corresponds to their maximum activity period of the day [58]. The total time spent in front or back position was pooled by sample points for each hive because hornets could not be differentiated. There were only 19 comparisons (instead of 22) because there was no hornet at 02∶00 pm in three sample points (one for H1 and two for H2).

*Apis mellifera* individuals being more numerous than hornets, their behaviour was monitored the first two minutes of each hour to score the number of honeybees flying in and out the hive. We used the number of flying honeybees as a parameter of the whole colony activity. Videos were visualized using VLC software (v. 1.1.11) and analyzed by a single person (LL).

The parameters we monitored in the videos allowed: 1) to compare the time spend by the hornets in front or back position with respect to the hive at 02∶00 pm, 2) to determine which honeybees (flying in or out the hive, on the hive walls or on the ground) were overall the more prone to predation by *V. velutina*, 3) to analyse the relation between the number of hornets and the number of caught honeybees, 4) to describe the diurnal and seasonal variations of the hornet and the honeybee activity as well as the evolution of the number of captures.

### Statistical Analyses

The total time spent in stationary flight by *V. velutina* in front or back position to the hive was compared using Wilcoxon test. Overdispersed Poisson log-linear Generalized Linear Model (GLM) was first used to analyse the location of honeybee when caught by a hornet (i.e., flying in and out the hive, on the ground, on the hive, at the hive entrance), associated with a Wilcoxon pairwise multiple comparison tests implemented with the Benjamini-Yekutieli’s correction [59]. Poisson models corrected for overdispersion were preferred to classical Poisson models based on overdispersion tests [60]. The statistical significance of each parameter was tested with likelihood ratio-based *χ* ^2^-statistics for unbalanced design [61]. Then, we only retained the number of individuals entering the hive to represent honeybee flying activity because there was no difference between the number of honeybee leaving and entering for both H1 and H2 (Wilcoxon tests, H1: *Z = *2528, *P* = 0.26, *n* = 139 and H2: *Z* = 2809, *P* = 0.24, *n* = 139). The relation between the number of hornets and the number of honeybees caught was also tested with a GLM, including a quadratic effect of the number of hornets to account for potential maximum effect. The diurnal and seasonal variations of (*i*) *V. velutina* predation pressure, *(ii)* the number of honeybee captures, and *(iii) A. mellifera* flying activity were also described using GLMs. In these models, the diurnal and seasonal effects were also included as quadratic effects to account for cyclic activities. All GLMs included a hive effect to account for potential differences between honeybee colonies.

All statistics were done with R software (v. 2.10.1 [62]) implemented with the following packages: *epicalc* for overdispersion detection; *dispmod* for fitting overdispersed Poisson log-linear GLMs, and *car* for deviance analysis for unbalanced design.

## Results

### Stationary Flight Position of *Vespa velutina* Workers

*Vespa velutina* workers stayed generally ten times longer in front than in back position (1^st^ quartile<median <3^rd^ quartile – in seconds, face: 10.00<97.00<152.50 and back: 1.50<8.00<13.50, Wilcoxon test: *Z* = 188.5, *P*<0.001, *n*_ sample points_ = 19, 51 and 41 hornets for H1 and H2 respectively).

### Overall Preference on Prey Location at Catching by *Vespa velutina*

**Figure 2:**
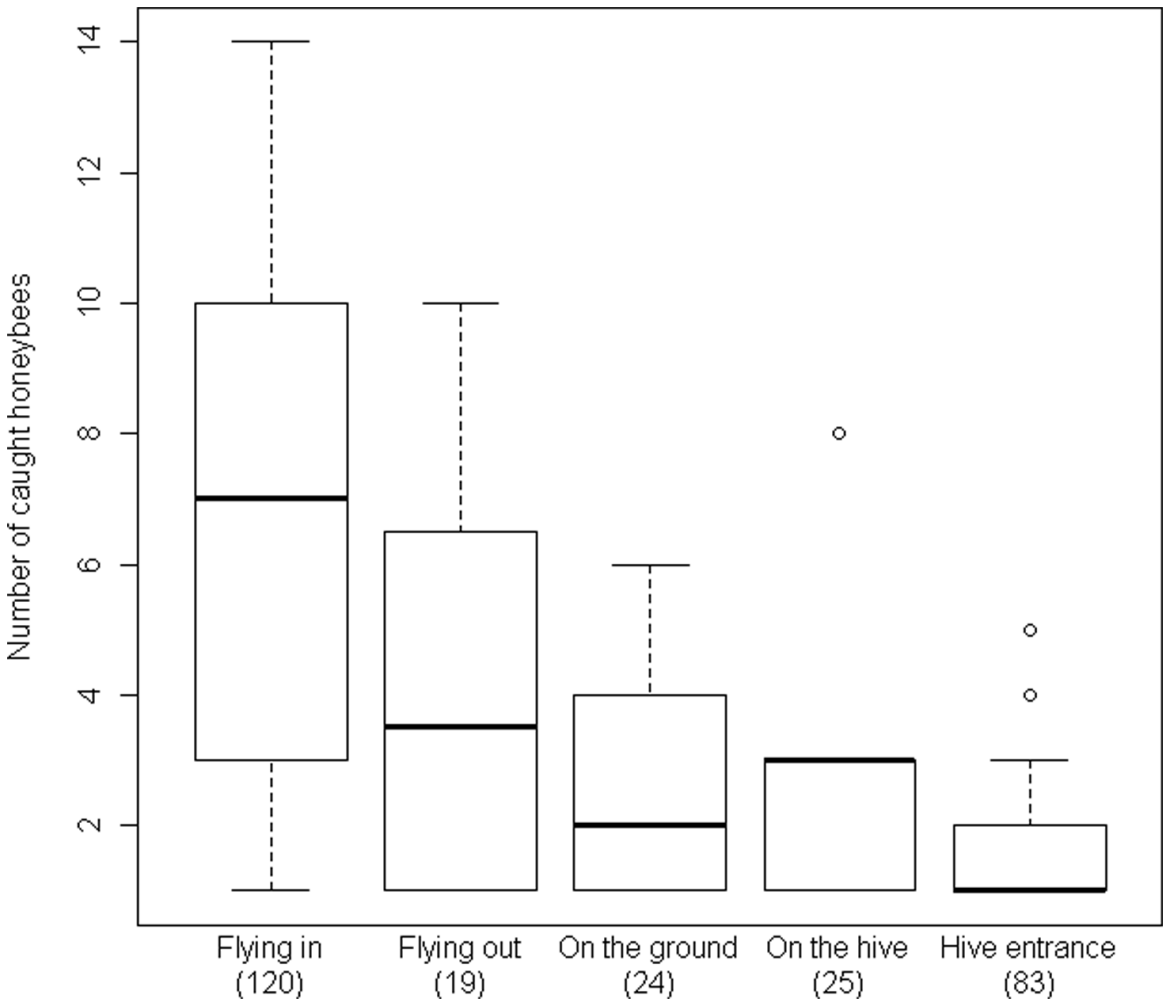
Distribution of the honeybees caught by *Vespa velutina*. The data are pooled for H1 and H2. Boxes, plain line, dashed lines, and open circles represent 50% of all values, medians, 1.5 interquartile range and extreme values respectively. Sample sizes for each location are presented in parentheses.

The number of honeybees caught differed only between their locations at the hive (GLM Poisson family: *χ* ^2^ = 25.81, *df* = 4, *P*<0.0001, Fig. 2) but not between hives (hive effect: *χ* ^2^ = 0.23, *df* = 1, *P* = 0.63, nor interaction hive x location: *χ* ^2^ = 1.58, *df* = 4, *P* = 0.81). This difference was solely due to the fact that flying honeybees returning to the hive suffered more predation than those guarding the hive entrance (Wilcoxon multiple comparison tests: *P* = 0.01), all others comparisons being non-significant (all *P*>0.20, Fig. 2).

### Relation between the Number of *Vespa velutina* and the Number of *Apis mellifera* Caught

**Figure 3:**
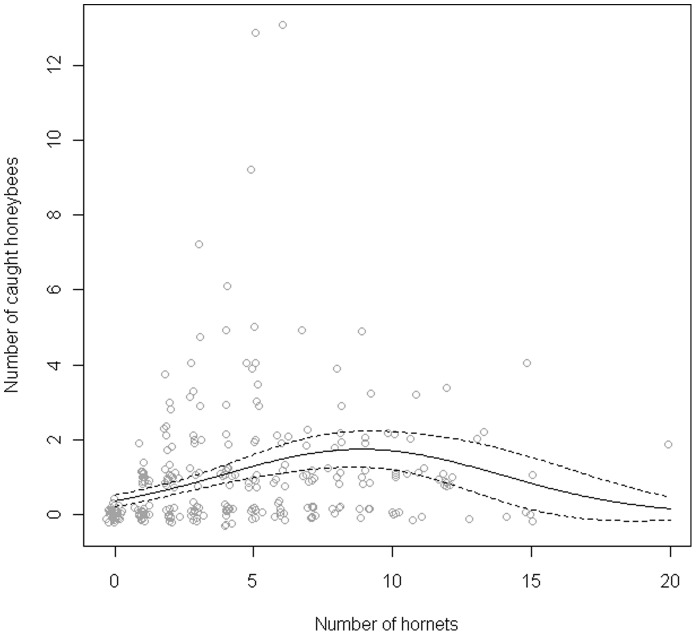
Relation between the number of *Vespa velutina* and the number of *Apis mellifera* caught. The data are pooled for H1 and H2. Predicted values fitted with the GLM model (plain line) with 95% confidence interval (dash lines).

The number of honeybee captures, assessed by the number of honeybees caught by hornets during five minutes, did not differ between hives (GLM Poisson family: *χ* ^2^ = 0.35, *df = *1, *P* = 0.55) but depended on the number of hornets (linear effect: *χ* ^2^ = 27.04, *df* = 1, *P*<0.0001 and quadratic effect: *χ* ^2^ = 17.58, *df* = 1, *P*<0.0001, Fig. 3) with a similar pattern on both hives (interaction with linear effect: *χ* ^2^ = 0.40, *df* = 1, *P* = 0.53, and interaction with quadratic effect: *χ* ^2^ = 0.52, *df* = 1, *P* = 0.47). The number of captures increased with the number of chasing hornets, reaching a maximum when nine hornets per hive were observed and decreasing for a higher number of individuals (Fig. 3).

### Diurnal and Seasonal Variations of *Vespa velutina* Predation Pressure

**Figure 4:**
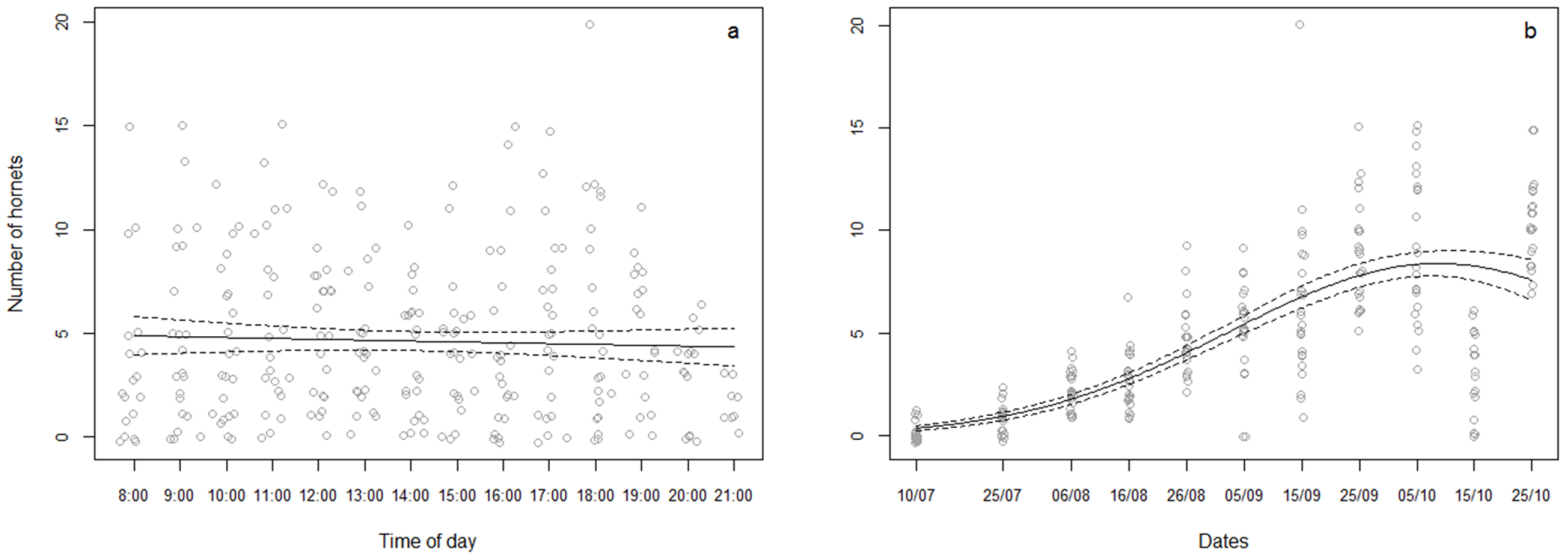
Variation of the number of *Vespa velutina* during a) the day and b) the season. The data are pooled for H1 and H2. Predicted values fitted with the GLM model (plain line) with 95% confidence interval (dash lines).

*Vespa velutina* predation pressure, assessed by the maximum number of hornets observed at the same time during five minutes, was similar on H1 and H2, did not vary during the day but during the season: the number of *V. velutina* increased from July to early October and then decreased (Table 1, Fig. 4). The small number of hornets observed on 15^th^ October was due to unfavourable wind conditions.

**Table 1:** Summary of the GLM (Poisson family) of the diurnal and seasonal variation of *Vespa velutina* predation pressure.

	?^2^	df	*P*
Hive	2.45	1	0.12
**Date**	**206.84**	**1**	**<0.0001**
**Date^2^**	**96.27**	**1**	**<0.0001**
Hour	0.42	1	0.52
Hour^2^	0.62	1	0.43
Hive×Date	1.75	1	0.19
Hive×Date^2^	1.97	1	0.16
Hive×Hour	0.04	1	0.83
Hive×Hour^2^	0.05	1	0.82
Date×Hour	0.47	1	0.49
Date×Hour^2^	0.23	1	0.63
Date^2^×Hour	0.54	1	0.46
Date^2^×Hour^2^	0.28	1	0.60
Hive×Date×Hour	0.95	1	0.33
Hive×Date×Hour^2^	1.10	1	0.29
Hive×Date^2^×Hour	1.29	1	0.26
Hive×Date^2^×Hour^2^	1.42	1	0.23
Residuals		260	

Significant effects are in bold. The seasonal and diurnal effects appear as both linear (Date and Hour) and quadratic effect (Date^2^ and Hour^2^).

### Diurnal and Seasonal Variations of *Apis mellifera* Flying Activity

**Figure 5:**
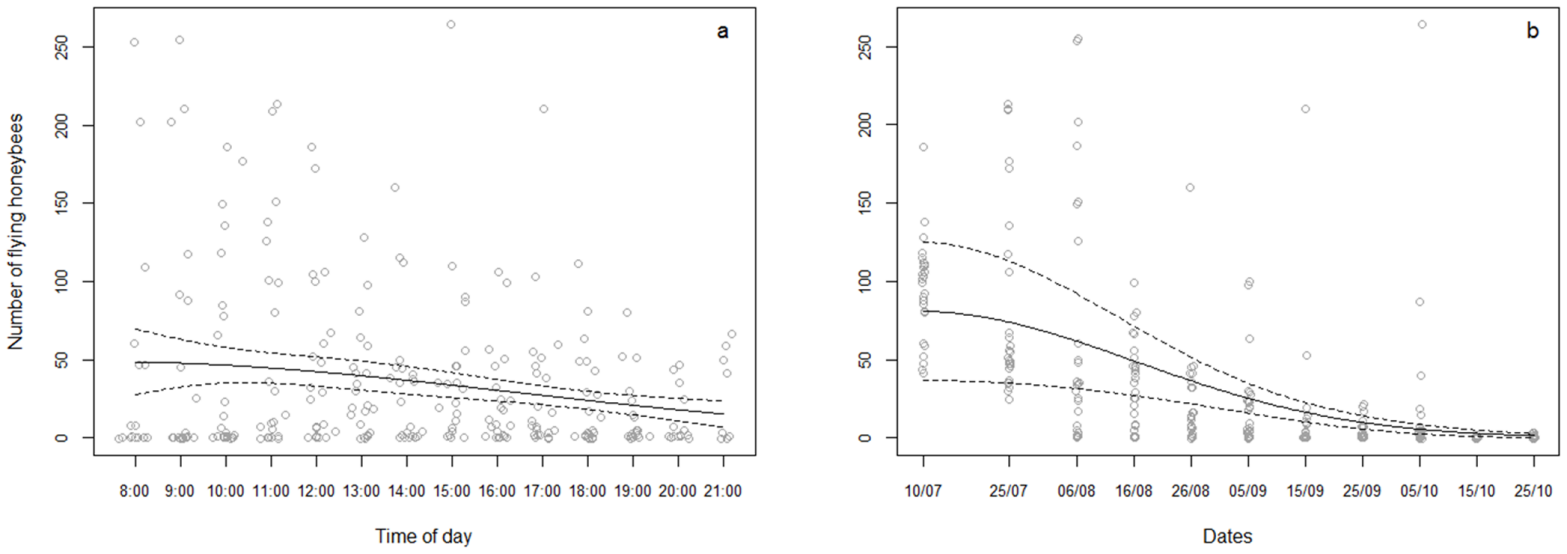
Variation of the number of flying *Apis mellifera* during a) the day and b) the season. The data are pooled for H1 and H2. Predicted values fitted with the GLM model (plain line) with 95% confidence interval (dash lines).

The flying activity of *A. mellifera*, assessed by the number of honeybees entering the hive during two minutes, was similar in H1 and H2 which allows considering these two hives as equivalent and varying during the day and the season (Table 2). During the day, the number of honeybees was higher early in the morning and then decreased in the afternoon and evening (Fig. 5a). At the seasonal time scale, the flying activity was higher in July and decreased throughout the summer until October (Fig. 5b).

**Table 2:** Summary of the GLM (Poisson family) of the diurnal and seasonal variation of *Apis mellifera* flying activity during *Vespa velutina* predation.

	?^2^	df	*P*
Hive	1.013	1	0.314
Date	0.530	1	0.467
**Date^2^**	**22.092**	**1**	**<0.0001**
**Hour**	**58.400**	**1**	**<0.0001**
**Hour^2^**	**58.464**	**1**	**<0.0001**
Hive×Date	0.001	1	0.977
Hive×Date^2^	0.005	1	0.942
Hive×Hour	0.106	1	0.745
Hive×Hour^2^	0.094	1	0.759
Date×Hour	0.522	1	0.470
Date×Hour^2^	0.885	1	0.347
Date^2^×Hour	2.844	1	0.092
Date^2^×Hour^2^	1.947	1	0.163
Hive×Date×Hour	1.928	1	0.165
Hive×Date×Hour^2^	1.597	1	0.206
Hive×Date^2^×Hour	2.913	1	0.088
Hive×Date^2^×Hour^2^	2.420	1	0.120
Residuals		259	

Significant effects are in bold. The seasonal and diurnal effects appear as both linear (Date and Hour) and quadratic effect (Date^2^ and Hour^2^).

### Diurnal and Seasonal Variations of the Number of Honeybee Captures

**Figure 6:**
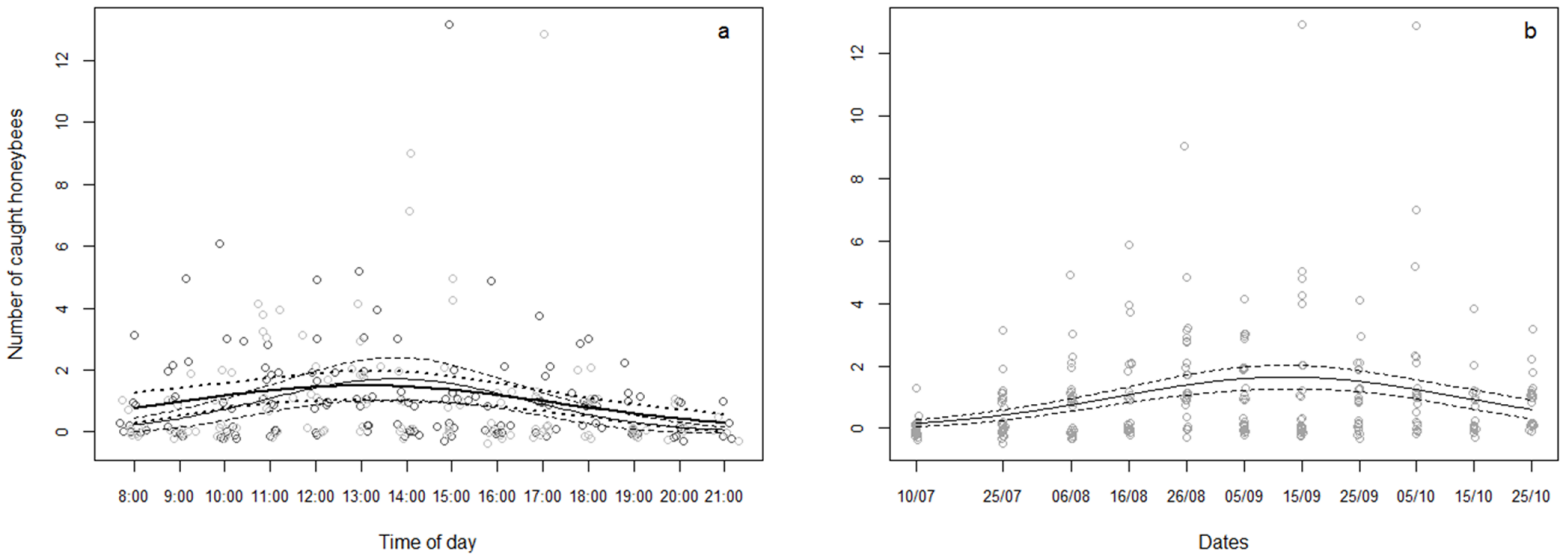
Number of honeybees caught by *Vespa velutina* during a) the day and b) the season. For (a), H1 data are in bold line/dark grey dots and H2 data are in thin line/light grey dots. For (b), the data are pooled for H1 and H2. Predicted values fitted with the GLM model (plain line) with 95% confidence interval (dash lines).

The number of honeybee captures, assessed by the number of honeybees caught by hornets during five minutes, varied during the day with different pattern between hives (Table 3). First, it increased during the morning with a slight difference between H1 and H2, the maximum number of captures being reached between 01∶00 and 02∶00 pm, and then, it decreased during the afternoon (Fig. 6a). The number of honeybees caught by *V. velutina* also varied seasonally with no difference between hives (Table 3). The number of honeybee captures increased from July to mid September and then decreased (Fig. 6b).

**Table 3:** Summary of the GLM (Poisson family) of the diurnal and seasonal variation of the number of honeybee captures.

	?^2^	df	*P*
Hive	1.551	1	0.213
**Date**	**52.230**	**1**	**<0.0001**
**Date^2^**	**46.798**	**1**	**<0.0001**
**Hour**	**29.185**	**1**	**<0.0001**
**Hour^2^**	**31.862**	**1**	**<0.0001**
Hive×Date	0.651	1	0.420
Hive×Date^2^	0.765	1	0.382
**Hive**×**Hour**	**5.289**	**1**	**0.021**
**Hive**×**Hour^2^**	**5.109**	**1**	**0.024**
Date×Hour	0.056	1	0.812
Date×Hour^2^	0.083	1	0.773
Date^2^×Hour	0.197	1	0.657
Date^2^×Hour^2^	0.084	1	0.772
Hive×Date×Hour	0.010	1	0.919
Hive×Date×Hour^2^	0.029	1	0.864
Hive×Date^2^×Hour	0.001	1	0.978
Hive×Date^2^×Hour^2^	0.001	1	0.978
Residuals		260	

Significant effects are in bold. The seasonal and diurnal effects appear as both linear (Date and Hour) and quadratic effect (Date^2^ and Hour^2^).

## Discussion

### *Vespa velutina* Predation Behaviour on Hives

*Vespa velutina* hunts by hovering facing the hive entrance (bee-hawking), to catch honeybees at any time during the day. Overall, flying honeybees paid the higher tribute compared to the others honeybees located close to the hive. Most of the flying honeybees are foragers and the fact that they are more prone to predation by *V. velutina* can be due to their pollen or nectar loads which can represent up to 40% extra body mass (see [63] and references therein), thus reducing their flying manoeuvrability to escape the predator. It may also result from the fact that they are the oldest individuals within the colony [64], [65]. Indeed, they may exhibit more wing damages than younger individuals which alter their flying ability [66], [67]. Interestingly, the number of captures reached a maximum for an intermediate number of hornets (nine hornets) in front of each hive (Fig. 3). Two non-mutually exclusive hypotheses might explain this pattern. *First*, it could result from the competition between hornets. Such interactions have been previously described in *V. multimaculata* and *V. orientalis* hunting *A. nuluensis* and *A. mellifera* respectively [68], [69]. In both cases, individuals exhibit intra-specific agonistic behaviours and the time spent in such interactions reduces their impact on the prey. *Second*, we may also consider the presence of individuals which do not forage on the site. Indeed, although most of *Vespa* species are solitary foragers, *V. mandarinia*, the giant Japanese hornet, develops group predation. During the pillage of a prey nest, some *V. mandarinia* guard the site night and day in order to exclude non-nestmates [70]. A capture-mark-recapture study has shown that some *V. velutina* individuals are mainly present in the vicinity of one specific hive and may possibly guard their foraging site (K. Monceau and D. Thiéry, unpublished data) and thus would not participate in honeybee captures. However, conclusions cannot be drawn from the data that we have obtained since hornets were not individually marked. Thus, individuals engaged in intra-specific agonistic behaviours or potential patrollers cannot be reliably identified. New experiments need to be planned to address this specific question.

### Prey and Predator Patterns of Activity

The predator daily rhythm may be driven by its own biological rhythm or by the activity of its prey, i.e., its availability [25]–[27]. At the seasonal time scale, the number of *V. velutina* increased during the summer until October. Similarly, the number of honeybee captures also increased from July to mid-September. These results are congruent with our previous study on the predation pressure dynamics of *V. velutina* showing that this increase reflects the hornet population dynamics [57]. During the day, the number of *V. velutina* did not vary whereas *A. mellifera* flying activity decreased suggesting that hornet occurrence at the hive does not depend on honeybee activity but might be an intrinsic property of the predator. However, according to our results, *V. velutina* individuals are more efficient predators at midday sun. Indeed, at this time, the number of honeybee captures was at its maximum while the number of *V. velutina* did not increase and was not related to any variation in *A. mellifera* flying activities. Like most of insect species, Vespidae foraging activities are influenced by climatic conditions [71]–[73]. We showed in a previous study that *V. velutina* predation was driven by the direct effect of wind, and also by the seasonal effect of both temperature and humidity [57]. This improved performance could result from the temperature increase at midday sun or to a higher level of solar irradiation. Indeed, an increased activity during noon-hours has already been described in *V. orientalis* and attributed to the UVB irradiation variations [74], [75]. Recent works have compared the cuticle of *V. orientalis* to photovoltaic cells converting solar energy into metabolic energy for flight muscles [74], [76]. Such accumulation could facilitate stationary flight but also flight speed and acceleration performances thus improving the hunting efficiency.

### Native Prey and Alien Predator: between Naïveté and Lack of Efficacy

In Europe, *A. mellifera* is also confronted to a lesser extend to the predation on hives by the native hornet species, *V. crabro* [77] which behaves similarly to *V. velutina* as both species bee-hawk in front of hives [16], [77]. Thus, *A. mellifera* might quickly respond by recognizing *V. velutina* as a predator since it belongs to the same predator archetype as *V. crabro* [41], [42]. Therefore, honeybees may not be considered a completely naïve prey and colonies may be able to adjust their behaviour to this kind of predation risk, even though *V. crabro* predation is far less intense. Indeed, the number of *V. crabro* in apiaries is often very low; *V. crabro*: *V. velutina* ratios of 1∶40 to 1∶70 were counted in our study area (K. Monceau and D. Thiéry, unpublished data). The observed prey may thus be globally considered not adapted to the predation exerted by *V. velutina*. In such a predation, individual defence that may be sufficient against *V. crabro* would not be sufficient against *V. velutina*. Consequently, at an early stage of the co-evolutive process between a native-naïve prey and an invasive predator, it is not surprising to find that the local honeybees do not respond adequately to *V. velutina* predation pressure. However, the number of hornets recruited for hunting honeybees is rather constant through the day, even though the maximal efficacy is only at midday sun. Our results also suggest that the apparent lack of efficacy of *V. velutina* to exploit its prey is due to constraints from its own behaviour (intra-specific competition) and biology (use of solar radiation). These constraints could be favourable to honeybees since the pressure exerted by *V. velutina* would be higher without such limitations. Moreover, the honeybee activity decreased through the day. Although it may be a consequence of *V. velutina* predation, it may also represent a strategy for honeybee colonies to maximise activities outside the hive during the morning hours, i.e., when hornets are less efficient. Indeed, honeybees are able to assess predation risk in food patches and to communicate this risk to their nestmates [78], [79]. Thus, the behaviour of the colony could be adjusted in accordance with the information shared by flying honeybees which have experienced and escaped from hornet attacks. In the absence of efficient defensive behaviours, such an adjustment may prevent from colony collapse.

### Conclusion

In this biological invasion, *V. velutina* represents a novel source of stress to honeybee colonies. However, the impact of *V. velutina* is limited by its own biology and behaviour and did not match the pattern of activity of its prey. Usually, native prey-alien predator interaction studies mainly focus on the unfit antipredator behaviour of the naïve prey but finally, our results suggests that the behaviour of the alien predator may also be unfit. In the present case, at the early stage of the invasion process, *V. velutina* predation pressure could have been much higher if not limited. Conversely, this behaviour may also represent a major advantage for the colonising process. This limited impact on honeybee colonies may also limit food depletion and thus facilitate *V. velutina* expansion in providing resources during its entire life cycle. From the prey point of view, these limits may represent an “open-window” that the honeybees may use to limit predation pressure, antipredator responses being potentially rapidly selected [35].
